# pH-dependent endocytosis mechanisms for influenza A and SARS-coronavirus

**DOI:** 10.3389/fmicb.2023.1190463

**Published:** 2023-05-10

**Authors:** Amar Aganovic

**Affiliations:** Faculty of Engineering Science and Technology, UiT The Arctic University of Norway, Tromsø, Norway

**Keywords:** respiratory virus, influenza A, SARS-coronavirus, pH, endocytosis, enveloped virus

## Abstract

The ongoing SARS-CoV-2 pandemic and the influenza epidemics have revived the interest in understanding how these highly contagious enveloped viruses respond to alterations in the physicochemical properties of their microenvironment. By understanding the mechanisms and conditions by which viruses exploit the pH environment of the host cell during endocytosis, we can gain a better understanding of how they respond to pH-regulated anti-viral therapies but also pH-induced changes in extracellular environments. This review provides a detailed explanation of the pH-dependent viral structural changes preceding and initiating viral disassembly during endocytosis for influenza A (IAV) and SARS coronaviruses. Drawing upon extensive literature from the last few decades and latest research, I analyze and compare the circumstances in which IAV and SARS-coronavirus can undertake endocytotic pathways that are pH-dependent. While there are similarities in the pH-regulated patterns leading to fusion, the mechanisms and pH activation differ. In terms of fusion activity, the measured activation pH values for IAV, across all subtypes and species, vary between approximately 5.0 to 6.0, while SARS-coronavirus necessitates a lower pH of 6.0 or less. The main difference between the pH-dependent endocytic pathways is that the SARS-coronavirus, unlike IAV, require the presence of specific pH-sensitive enzymes (cathepsin L) during endosomal transport. Conversely, the conformational changes in the IAV virus under acidic conditions in endosomes occur due to the specific envelope glycoprotein residues and envelope protein ion channels (viroporins) getting protonated by H+ ions. Despite extensive research over several decades, comprehending the pH-triggered conformational alterations of viruses still poses a significant challenge. The precise mechanisms of protonation mechanisms of certain during endosomal transport for both viruses remain incompletely understood. In absence of evidence, further research is needed.

## 1. Introduction

During the first two decades of the 21st century, humanity has faced significant difficulties due to the emergence of highly pathogenic and contagious respiratory viruses, including severe acute respiratory syndrome coronavirus (SARS-CoV), Middle East respiratory syndrome coronavirus (MERS-CoV), IAV virus subtype H1N1, and the current severe acute respiratory syndrome coronavirus 2 (SARS-CoV-2). The substantial levels of illness (Shi et al., 2017; James et al., 2018; Lopez-Leon et al., 2021), increased mortality (Zucs et al., 2005; Iuliano et al., 2018; Hansen et al., 2022; Msemburi et al., 2023), and significant socioeconomic consequences (Fendrick et al., 2003; Cutler and Summers, 2020) caused by these respiratory viruses have emphasized the need for effective measures to contain their spread. To mitigate the transmission of these viruses, both occupational and public health measures have been implemented, such as physical distancing, mask-wearing, disinfection, and the development of antiviral drugs, antibody-based therapies, and vaccines. In addition, since the recognition of airborne transmission as the main route of spread, engineering strategies have recommended the enhancement of indoor air quality through improved ventilation, air purifiers, and/or filtration of recirculated air (Sachs et al., 2022). Other approaches have also sought to take advantage of the sensitivity of enveloped viruses to environmental conditions, including temperature (Dabisch et al., 2020; Biryukov et al., 2021), UV levels (Dabisch et al., 2020; Biasin et al., 2021), and relative humidity (Dabisch et al., 2020). However, one environmental factor that has received limited attention during the pandemic is the potential impact of the pH value of the virus aerosol microenvironment (Luo et al., 2023).

Compared to the limited knowledge on the pH impact on viral survival in extracellular environments including aerosols and surfaces, inquiry into the impact of pH on respiratory viruses in intracellular environments began decades ago (Maeda et al., 1981). Both influenza and coronaviruses are known to be sensitive to changes in pH during the endocytosis process, which is a critical step in the infection cycle by which viruses enter host cells from the extracellular environment. For example, it is well-accepted that low pH induces viral disassembly and promotes the release of genetic material within the host cell (Stegmann et al., 1987). When the pH within the host cell becomes acidic (i.e., the concentration of hydrogen ions increases), the protein residues of the lipid membrane may become protonated, which eventually induces conformational changes in the envelope glycoproteins to a large extent, and to a lesser extent, in the viroporins (Caffrey and Lavie, 2021). This can cause the envelope to become more permeable, which may allow ions and other molecules to enter the virus and disrupt its structure. Additionally, changes in pH can also affect the electrostatic interactions between the viral proteins and the envelope, further destabilizing the virus (Shtykova et al., 2017). The COVID-19 pandemic has reignited interest in this topic, and recent studies have tried to elucidate the mechanisms involving pH-induced changes in the envelope disassembly of SARS-CoV-2 (Kreutzberger et al., 2022; Luo et al., 2023). In addition, the technological advancements in microscope technologies have enabled scientists to gain new insights into the mechanisms by which viruses are affected by changes in pH during endocytosis (Assaiya et al., 2021; Guaita et al., 2022).

In this review, I discuss developments in our understanding of the pH-induced endocytosis mechanisms in IAV and SARS-coronavirus. Drawing upon extensive literature from the last few decades and latest research, this mini-review provides a detailed explanation of the pH-dependent structural changes necessary for viral disassembly and fusion during endocytosis. Using this summary, I recognize the significant differences that result in pH-induced structural changes preceding the viral disassembly and initiating fusion with the host cell. The main aim of this review is to provide a better understanding of how the structural changes of two the highly contagious enveloped respiratory viruses differ in response to intracellular acidic environments.

## 2. Influenza A

Influenza is a negative-strand RNA virus with eight ribonucleoprotein particles (RNPs) contained within a lipid envelope derived from the host plasma membrane. The IAV viral envelope contains two major glycoproteins proteins, hemagglutinin (HA) and neuraminidase (NA), that project from the lipid membrane as spikes (Sriwilaijaroen and Suzuki, 2012). A third envelope protein is a homotetrameric protein 2 (AM2) consisting of an extracellular N-terminal segment, a transmembrane segment, and an intracellular C-terminal segment (Pielak and Chou, 2011). The lipid membrane envelope encapsulates the M1 protein, consisting of the N-terminal domain, middle domain, and C-terminal domain, which forms a rigid matrix layer under the lipid envelope and interacts with both the viral RNP particles and lipid envelope with the cytoplasmic tails of HA and NA (Lamb and Choppin, 1983; Bouvier and Palese, 2008; Dou et al., 2018).

### 2.1. IAV pH-regulated endocytic pathways: clathrin-mediated endocytosis

**FIGURE 1:**
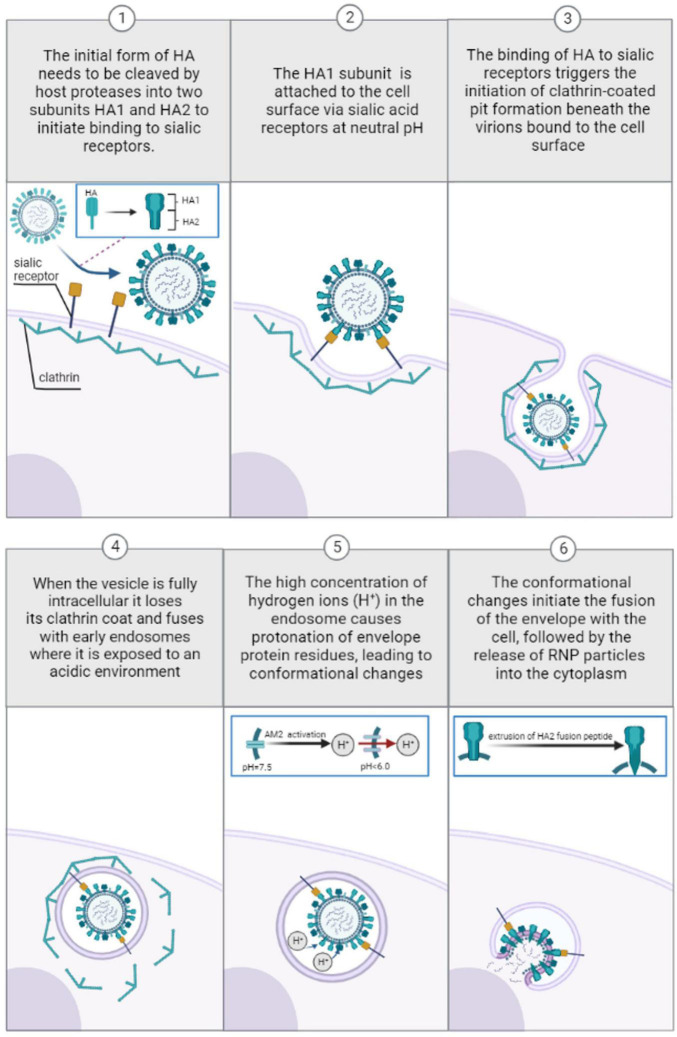
Pre-viral entry of Influenza virus and binding to sialic acid receptors during clathrin-mediated endocytosis at neutral pH (1–3). Viral entry and transport through endosomes during clathrin-mediated endocytosis: low pH-induced conformational changes (4–6).

There are several types of viral entries to the cell, also known as endocytic pathways that can be utilized by influenza viruses, including predominantly clathrin-mediated and caveolin-mediated endocytosis (Lakadamyali et al., 2004; Brandenburg and Zhuang, 2007; de Vries et al., 2011). The pathways differ in the manner by which the virus particle attaches to the surface of the host cell. Caveolin-mediated endocytosis does not require sialic acid receptors for internalization of the virus, unlike clathrin-mediated endocytosis (Lakadamyali et al., 2004). The trafficking of clathrin-mediated endosomes relies on acidic pH, while the transport of caveolae containing vesicles to the destination is a neutral pH selection (Kiss and Botos, 2009). Therefore, pH-independent caveolin-mediated endocytosis is not considered in this paper. Before attaching to sialic acid, the HA glycoprotein in the viral membrane initially exists as a single polypeptide known as HA0, which must be cleaved by the host’s trypsin and serine-like proteases (TMPRSS2) to form a complex consisting of three HA1 (positively charged) and three HA2 (negatively charged) polypeptide chains linked by two disulfide bonds (Chen et al., 1998). HA1, which is located distal to the virus membrane, is responsible for receptor binding. On the other hand, HA2, which is located proximal to the membrane, anchors HA in the envelope and contains the fusion peptide (Benton et al., 2020a). As shown on Figure 1, once the HA cleavage occurs, the receptor binding pocket at the top of the positively charged HA1 subunit becomes available for binding with negatively charged sialic-acid receptors (Mair et al., 2014a). After viral internalization, the incoming virus is transported through early endosomes (pH range ∼5.6–6.5) (Padilla-Parra et al., 2012), late endosomes (pH range ∼5.0–5.5) (Wallabregue et al., 2016), and lysosomes (pH range between 4.6 and 5.0) (Luzio et al., 2007).

Eventually, the cleaved pre-fusion neutral pH HA (Staschke et al., 1998) is attached to the cell surface via sialic acid receptors. According to experimental observations (Carr et al., 1997), the cleaved extracellular HA protein is trapped in a metastable state at neutral pH before viral entry. At neutral pH, molecular modeling studies have demonstrated that there is a strong electrostatic attraction between the positively charged HA1 and negatively charged HA2 monomers, while there is a repulsive electrostatic force between three subunits of either HA1 or HA2 (Huang et al., 2002). Consequently, electrostatic repulsion between the three HA1 monomers and three HA2 monomers is offset by electrostatic attraction between the HA1 and HA2 domains, thereby preserving a metastable trimeric association of the monomers. The binding of HA1 to sialic residues on membrane cells triggers the initiation of clathrin-coated pit formation beneath the virions bound to the cell surface (Rust et al., 2004). These pits then bud from the membrane to form small intracellular clathrin-coated vesicles containing the virions and their bound receptors (Matlin et al., 1981; Lakadamyali et al., 2004). When the vesicle is fully intracellular it loses its clathrin coat and ultimately fuses with early endosomes where it is exposed to an acidic environment (pH range ∼5.6–6.5) (Padilla-Parra et al., 2012). The high concentration of hydrogen ions (H+) in the endosome causes protonation of specific to envelope glycoprotein residues, leading to eventual conformational changes within the virus critical for later fusion with the host cell.

#### 2.1.1. pH-induced hemagglutinin conformational changes

The pH dependence of the early stages of HA conformational change is regulated by the histidine residue HR184 of the HA1 and HA2 monomers (Mair et al., 2014b; Trost et al., 2019). The side chain of histidine is uncharged at physiological pH (∼7.4) because it has a pKa of approximately 6. As the environmental pH decreases, the HA histidine residue with a pKa value greater than the environmental pH becomes protonated (Mair et al., 2014b; Trost et al., 2019). Protonation leads to a significant increase in positive net charge, inducing the repulsion of HA1 monomers (Huang et al., 2002) and the partial dissociation of HA1 globular domains (Zhou et al., 2014). Thus, protonation triggers the enlarging of the cleavage between HA1 and HA2 monomers. As a consequence, water can enter the central cavity, which in turn induces the structural transitions of the HA2 monomer/sequences (Kemble et al., 1992) which have originally been shielded from contact with water (Böttcher et al., 1999; Huang et al., 2002). Interaction with water induces extrusion of the HA-2 fusion peptide from its buried position in the HA trimer to the distal tip of the HA spike (Ruigrok et al., 1989), eventually triggering the disintegration of the viral membrane by forming a pore through which the genomic segments of the virus are released (Cross et al., 2009; Rice et al., 2022). An HA that is too acid stable may not be sufficiently sensitive to trigger fusion pH-dependent uncoating, meaning that acid stability could restrict a virus’ ability to replicate in intracellular environments. Measured HA activation pH values across all subtypes and species range from ∼5.0 to 6.0, trending higher in highly pathogenic H5N1 (Zaraket et al., 2013) and H7N9 strains (pH 5.6–6.0) (Chang et al., 2020) whereas seasonal human strains are more acid-stable (pH of fusion 5.0 to 5.6) (Galloway et al., 2013).

#### 2.1.2. pH-induced AM2 conformational changes

The AM2 protein forms a pH-activated proton-selective channel (Sakaguchi et al., 1996; Cady et al., 2009) essential for the acidification of the virus interior, thereby facilitating the dissociation of the matrix protein M1 from the viral nucleoproteins−a step that precedes fusion-pore formation (Zebedee and Lamb, 1988; Ivanovic et al., 2012). As the pH of the endosome encapsulating the virus is lowered, the AM2 channel becomes activated, allowing a unidirectional proton across the membrane to equilibrate the pH of the virus interior with that of the acidic endosome (Kelly et al., 2003). Once activated, the M2 channel conducts 10 to 10,000 protons per second (Mould et al., 2000a; Lin and Schroeder, 2001). This pH-activated protonation is mediated by an interplay between four proton-selective histidine residues His-37 and the proton conductive four tryptophan 41 (Trp 41) residues, both located in a narrow aqueous pore of the transmembrane domain (TMD) (Wang et al., 1995; Venkataraman et al., 2005; Hu et al., 2006; Schnell and Chou, 2008). Two proton conduction mechanisms have been proposed: the “water wire model” and the “proton relay model” mechanism. According to the “water-wire model,” the pore is essentially closed at neutral pH (pH = 7.5) as the Val 27 residue at the N terminal and the Trp 41 gate block water from freely entering into the pore, thus preventing proton diffusion across the membrane (Schnell and Chou, 2008). Lowering the pH protonates the imidazole rings of His-37, resulting in several imidazolium per channel, which repel each other and destabilize the transmembrane-helices packing. This conformational rearrangement breaks interactions between Trp 41 and Asp 44 residue and widens the pore, followed by a formation of a continuous hydrogen-bonded water network over which protons hop utilizing the Grotthuss mechanism (Chen et al., 2007). Carr–Purcell–Meiboom–Gill (CPMG) experiments have found that lowering the pH from 7.5 to 6.0, increases the frequency of Trp 41 gate opening by more than fourfold, while no significant frequency is changed when lowered to pH = 7.0 (Schnell and Chou, 2008). Under ideal conditions of the “water-wire model,” the constricted N-terminal only allows protons to penetrate the aqueous pore through a hydrogen-bonded water network. This assumption is confirmed by many electrophysiological studies that show that the highly selective M2 channel is virtually impermeable to Na^+^, K^+^, or Cl^–^ ions regardless of external pH conditions (Chizhmakov et al., 1996, 2003; Mould et al., 2000b; Lin and Schroeder, 2001; Intharathep et al., 2008). Accordingly, the M2 protein only transports protons, and this permeation increases tenfold as the pH drops from below 8.5 until it reaches a saturation level close to pH = 4 (Chizhmakov et al., 1996). The selectivity, however, may not be absolute as the permeation of other ions through this channel has been suggested from earlier experiments (Pinto et al., 1992). It has been suggested that experimental artifacts from earlier studies are responsible for these differences (Chizhmakov et al., 1996).

On the other hand, the “proton-relay model” mechanism requires at least one non-protonated histidine at the gating region, with its two nitrogen atoms facing the extracellular side (Pinto et al., 1997; Chen et al., 2007). This model hypothesizes that one His-37 imidazole nitrogen atom is protonated by the entering hydronium ion before the other imidazole nitrogen releases its proton to the interior of the virus. Finally, the process is completed by flipping of imidazole rings, or tautomerization, to establish the original configuration to prepare for the next proton relay. The reliability of the model is uncertain as it appears that for a His residue to act as a proton relay two nitrogens from the same histidine residue must be exposed to water within the channel pore (Pinto et al., 1997). However, simulation studies have not revealed this specific conformation of His-37 (Phongphanphanee et al., 2010).

#### 2.1.3. pH-induced neuraminidase conformational changes

Neuraminidase (NA)’s primary role is in the later stages of infection, where it aids in the detachment and spread of the virus to new cells by removing sialic acids from cellular receptors and newly synthesized HA and NA on nascent virions (Palese et al., 1974; Basak et al., 1985). This process prevents the virus from binding back to the dying host cell and enables the efficient release of RNA genomes (Palese et al., 1974). NA is most effective at a pH range of 5.5–6.0 (Mountford et al., 1982; Lentz et al., 1987; McKimm-Breschkin, 2000), although certain viruses have been found to maintain stable NA activity at a lower pH range of 4.0–5.0, resulting in enhanced replication kinetics (Takahashi and Suzuki, 2015).

#### 2.1.4. pH-induced M1 conformational changes

The M1 protein binds both to the RNP complex and the lipid membrane. The M1–lipid binding is mediated through electrostatic interactions between the positively charged N-terminal domain residues (Arg76 and Arg78) and negatively charged cytoplasmic tails of HA and NA (Höfer et al., 2019), while the matrix protein interacts with the viral RNP complex inside the virus via the C-terminal domain (Shtykova et al., 2017) but also the middle domain (Noton et al., 2007). It is hypothesized that the acidification in the endosome causes the M1 protein to undergo a conformational change which ultimately allows the disassembly of the RNP-M1-lipid membrane complex and RNP detachment from the membrane (Calder et al., 2010; Fontana et al., 2012). Research by cryo-electron tomography (ET) further showed that the intermolecular interactions in the M1 layer are affected when the virions were incubated at pH 5.0, and the matrix layer was no longer seen in the virions (Lee, 2010). Specifically, other cryo-ET studies have indicated that acidification affects the oligomerization state of the M1 protein; it has been demonstrated that intact M1 display multiple-ordered forms of oligomers at neutral pH 7.4 which are dissociated at pH 5.0 (Zhang et al., 2012). Not until recently has the first full structure of full-length M1 been observed; subtomogram observations showed it contains a five histidine residues cluster that may serve as the pH-sensitive disassembly switch (Peukes et al., 2020). Despite these findings, the precise mechanism of protonation of the M1 protein during endosomal transport remains poorly understood, and further research is needed (Selzer et al., 2020).

## 3. SARS-coronavirus

The positive-stranded RNA genome of SARS-coronavirus encodes three membrane proteins: the spike (S) glycoprotein, responsible for binding the cell-surface receptor to induce virus-host cell fusion (Huang et al., 2020); and the viral envelope proteins consisting of the membrane (M) glycoprotein and the envelope (E) protein. The S protein anchored in the viral membrane is a trimer with each protomer composed of S1 and S2 subunits non-covalently bound in the pre-fusion state (Örd et al., 2020).

### 3.1. pH-dependent infection routes: S protein conformational changes activated by cathepsin L during clathrin-mediated endocytosis

SARS-coronavirus entry into target cells starts with protease-induced preactivation of the S1/S2 cleavage (Peacock et al., 2021). The protease-activated cleavage is followed by S1 binding to the host cellular receptor angiotensin-converting enzyme 2 (ACE2) (Li et al., 2003). Successive ACE2 binding further weakens the protease-induced preactivation of the S1/S2 cleavage, followed by cleavage of the S2 unit to generate S2′ (Benton et al., 2020b). The S2′ fusion peptide is then liberated and eventually penetrated the host target cell, ultimately leading to the fusion of the viral and host cell membranes after which viral RNA is released into the cytoplasm, where it replicates (Walls et al., 2017; Benton et al., 2020b). The conformational changes in the S2 unit can be triggered by either the transmembrane serine protease TMPRSS2 (Tortorici et al., 2019) or lysosomal cysteine protease cathepsin L in the endosomal compartment following ACE2-mediated endocytosis (Hoffmann et al., 2020; Zhao et al., 2021). However, the timing and dynamics of these proteolytic cleavages differ for different coronavirus types. After protease-preactivation of the S1/S2 cleavage, SARS-CoV-2 can use mutually exclusive routes to penetrate cells: one fast TMPRSS2-mediated plasma membrane entry (10 min) and one slower (40–50 min) clathrin-mediated endocytosis where S2′ cleavage is performed by cathepsin L (Koch et al., 2021; Jackson et al., 2022). TMPRSS2 is active at the cell surface regardless of pH conditions (Koch et al., 2021; Jackson et al., 2022), unlike cathepsin L, which requires a low-pH environment typical of endolysosomes (Mohamed and Sloane, 2006; Koch et al., 2021; Jackson et al., 2022).

**FIGURE 2:**
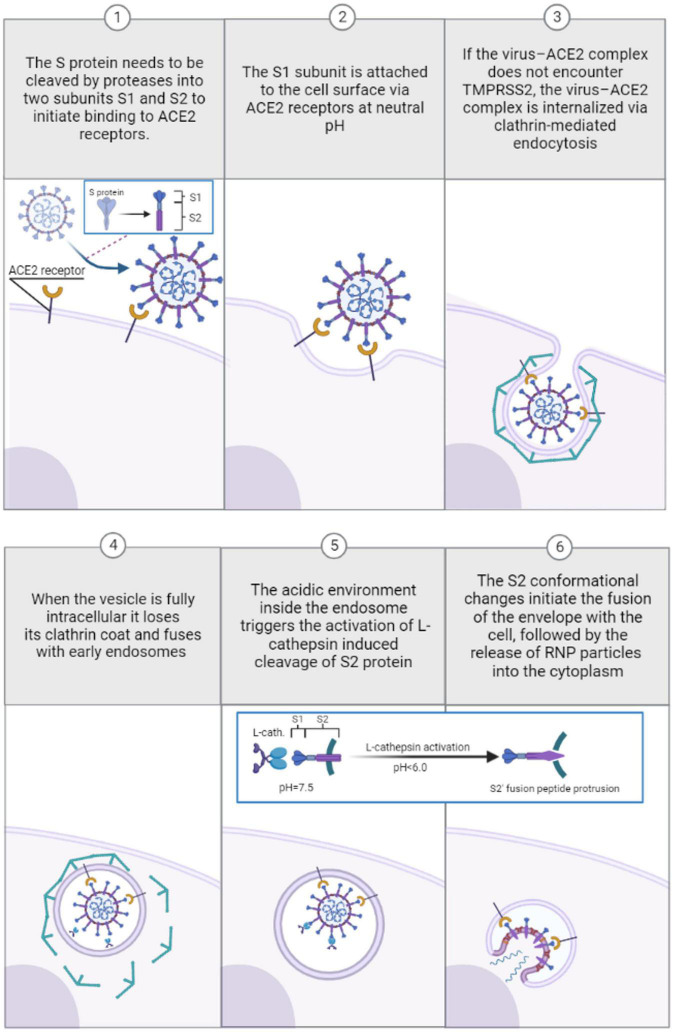
Pre-viral entry of SARS-coronavirus and binding to ACE-2 receptors during clathrin-mediated endocytosis in absence of TMPRSS2 (1–3). Viral entry and transport through endosomes during clathrin-mediated endocytosis: low pH-induced conformational changes induced by cathepsin L activation in acidified endosomes (4–6).

Thus, SARS-CoV-2 fusion is essentially independent of pH value as endosomal acid-dependent penetration through cathepsin L occurs only in cells devoid of TMPRSS2 (Koch et al., 2021) as shown in Figure 2. Although several studies support the view that TMPRSS2-dependent early entry route is more efficient and results in a more productive infection than the cathepsin L-activated mechanisms for some CoV strains (Shirato et al., 2017, 2018), other studies indicate that more recent Omicron SARS-CoV-2 variants favor the low-pH endosomal entry route (Meng et al., 2022). For fusion activity in SARS-CoV, cathepsin Ls have been shown to require reduced pH of at least 6.0 or lower, found in the endolysosomal compartment (Luzio et al., 2007; Padilla-Parra et al., 2012; Wallabregue et al., 2016). Human cathepsin L is very unstable (kinact = 0.15 s−1) at close to neutral conditions (pH = 7.4; 37°C) and the inactivation rates increase for at least one order of magnitude between pH 7.0 and 8.0 at 37°C [L109]. Interestingly, the cathepsin L activity is very temperature dependent: at pH = 7.4, a temperature rise from 5 to 37°C results in a thousand-fold increase in the inactivation rate (Turk et al., 1993). Bound to negatively charged surfaces, the cathepsin L activity also depends on the ionic composition of the exposed milieu (Dehrmann et al., 1995). Studies focusing on the composition of buffers have shown that different ionic solutions and ionic strengths have unique impacts on cathepsin L activity (Dehrmann et al., 1995, 1996). While in phosphate solutions the enzymatic activity occurs at a slightly acidic condition range of pH = 5.5–6.0 (Mason and Massey, 1992), the cathepsin L activity peaks at pH = 6.5 in acetate-MES2-Tris (AMT) buffers (Dehrmann et al., 1996) at constant molarity. In most enzyme-catalyzed reactions carried out in the laboratory the ionic strength is usually fairly high due largely to the high buffer concentrations needed to ensure constant pH. The higher the ionic strength, the greater the electrostatic interactions between ions in the solution, which can affect various chemical and physical properties of the solution (Kennedy, 1990). For instance, when increasing the ionic strength of a weak acid its optimum pH shifts to lower pH values at constant molarity, and the opposite trend is true for weak bases (Dennison, 2003). It has been observed that cathepsin L activity time is reduced with an increase in the ionic strength of phosphate buffer–a weak acid (Kennedy, 1990), implying increasing the ionic strength in weak acids may increase the optimum pH activity range for cathepsin L and vice versa. The effects of ionic strength on the activities of cathepsin Ls may be of key significance in establishing their true potential for extracellular activity processes such as anti-viral drug development (Turk et al., 1993; Dehrmann et al., 1995). While cathepsin L activity generally favors slightly acid conditions, in sodium citrate buffers cathepsin L is irreversibly inactivated at pH values lower than 4.0 (Turk et al., 1993), an acidic environment that can occur in intact cells where matured lysosomes may reach a pH as low as 3.8 (Berg et al., 1995; Van Dyke, 1995).

#### 3.1.1. pH-induced M protein conformational changes

The M protein is the most abundant structural protein and contains three transmembrane helices, with a short amino-terminal ectodomain and a large carboxy-terminal endodomain (Kuo et al., 2007). The M protein defines the envelope shape and is directly involved in virus assembly, replication, and membrane budding (Hu et al., 2003; Neuman et al., 2011). Although the structure of M protein forms a dimer that is structurally related to the SARS-CoV-2 ion channel ORF3a (Ouzounis, 2020; Dolan et al., 2022), reported cryo-electron microscopy structures revealed that is unlikely that the so far known forms of M protein function as an ion channel because its transmembrane region is highly hydrophobic and has no apparent ion permeation pathway (Zhang et al., 2022). These findings do not rule out the possibility that the two currently recognized forms of the M protein, an elongated and a compact one (Neuman et al., 2011; Dolan et al., 2022), represent closed conformations, and that a different, unknown form is responsible for ion conduction. Although the function of coronavirus M appears to be analogous to that of the virus M1 protein of influenza A (Neuman et al., 2011), no similar pH-sensitive behavior has yet been observed.

#### 3.1.2. pH-induced E protein conformational changes

The smallest of the major structural proteins, the multifunctional E protein acts on several aspects of the virus’ life cycle, including virus assembly, budding and pathogenesis (Schoeman and Fielding, 2019). The E protein is composed of a short hydrophilic N-terminal, followed by a large hydrophobic TMD, and ends with a long hydrophilic C-terminal domain, which compromises the majority of the protein Structurally, the TM domain of E forms a pH-sensitive pentameric ion channel (viroporin) on the ER/Golgi membrane, which is generally permeable to Ca2+, Na+, Mg2+ and K+ ions (Verdiá-Báguena et al., 2012; Nieto-Torres et al., 2015; Cabrera-Garcia et al., 2021; Xia et al., 2021), but also to H+ ions according to *in silico* studies and pH imaging (Cabrera-Garcia et al., 2021; Xia et al., 2021). In mammalian cells, the SARS-CoV-2 viroporin is activated at pH = 6.0 and 7.4, whereas at alkaline pH values of 8 and above the viroporin activity is reduced (Cabrera-Garcia et al., 2021; Xia et al., 2021). However, some inconsistencies between studies and tests have been noted. The varying ion channel characteristics of E proteins across different coronaviruses and even within different experimental settings have led to concerns that certain outcomes could be erroneous.

## 4. Discussion

In this review, I analyze and compare the conditions and mechanisms of pH-dependent endocytic pathways for IAV and SARS-coronavirus. While there are similarities in the pattern, there are notable differences in the mechanisms and incidence of pH-dependent endocytosis. Influenza viral entry primarily takes place via clathrin-mediated and caveolin-mediated endocytosis, but only the former is regulated by pH. After viral clathrin-mediated internalization, the incoming virion is transported through an acidic environment in early endosomes, late endosomes, and lysosomes. As the pH of the endosome encapsulating the virus is lowered, the virus structure undergoes several conformational changes. The lowered pH value induces the extrusion of the HA-2 fusion peptide from its buried position which forms a pore through which the genomic segments of the virus are released. In addition, the AM2 proton channel becomes activated, allowing a unidirectional proton across the membrane to equilibrate the pH of the virus interior with that of the acidic endosome. Lowered pH also induces conformational changes to the influenza NA and M1 protein, however, unlike HA and AM2 the precise mechanisms remain poorly understood, and further research is needed. Measured influenza activation pH values across all subtypes and species range from ∼5.0 to 6.0, trending higher in highly pathogenic H5N1 and H7N9 strains whereas seasonal human strains are more acid-stable (pH of fusion 5.0 to 5.6). Similar to IAV, SARS-CoV-2 can use mutually exclusive routes to penetrate cells: one fast pH-independent TMPRSS2-mediated plasma route, and one pH-dependent slow clathrin-mediated endocytosis via cathepsin L. The clathrin-mediated endocytosis through cathepsin L occurs only in cells devoid of TMPRSS2. Unlike TMPRSS2, which is active at the cell surface regardless of pH conditions, cathepsin L requires a low-pH environment typical of endolysosomes. Although several studies support the view that TMPRSS2-dependent early entry route is more efficient and results in a more productive infection than the cathepsin L-activated mechanisms for some SARS-coronavirus strains, recent studies indicate that Omicron SARS-CoV-2 variants favor the low-pH endosomal entry route. For fusion activity in SARS-coronavirus, cathepsin Ls have been shown to require reduced pH of at least 6.0 or lower. Although research has verified that the envelope E-protein serves as an ion channel when pH levels decrease, there have been certain inconsistencies observed between various studies and tests. Furthermore, unlike the influenza M1 protein, there has been no evidence of similar pH-sensitive behavior in the SARS-coronavirus M1 protein. However, studies also suggest that cathepsin L is irreversibly inactivated at pH values lower than 4.0, an acidic environment that may occur in matured lysosomes. The pH sensitivity of influenza A and SARS-CoV-2 viral glycoproteins is a potential target for therapeutic interventions and anti-viral drug treatments. So far, there is evidence suggesting that chloroquine, a weak base, inhibits the replication of those influenza A strains whose hemagglutinins require a low pH for their fusion activation (Di Trani et al., 2007). Chloroquine has shown potential in blocking the SARS-CoV-2 infection cycle by releasing basic side chains that raise the endosomal pH and inactivate cathepsin-L (Lan et al., 2022). However, the use of chloroquine for the treatment of COVID-19 triggered significant debate, especially since the drug is associated with side effects and exhibits only marginal efficacy (Chen et al., 2021; Kashour et al., 2021). With regard to alternative approaches for achieving endosomal deacidification, endosomal acidification inhibitors bafilomycin A1 and NH_4_Cl were shown to exert antiviral effects against SARS-CoV-2 *in vitro* cell models and *in vivo* in hACE2 transgenic mice, and thus should be evaluated as potential COVID-19 treatments (Shang et al., 2021). In summary: while both influenza and coronavirus may pursue pH-regulated endocytic pathways, the influenza virus does not require the presence of specific pH-sensitive enzymes (cathepsin L) during endosomal transport to activate fusion with the host cell. The review also notes that the precise mechanism of protonation mechanisms of certain envelope glycoproteins during endosomal transport for both viruses remains incompletely understood, and further research is needed.

## Author contributions

The author confirms being the sole contributor of this work and has approved it for publication.

## References

[B1] Assaiya A. Burada A. Dhingra S. Kumar J. (2021). An overview of the recent advances in cryo-electron microscopy for life sciences. *Emerg. Top. Life Sci.* 5 151–168. 10.1042/ETLS20200295 33760078

[B2] Basak S. Tomana M. Compans R. W. (1985). Sialic acid is incorporated into influenza hemagglutinin glycoproteins in the absence of viral neuraminidase. *Virus Res.* 2 61–68. 10.1016/0168-1702(85)90060-7 3984493

[B3] Benton D. Gamblin S. Rosenthal P. Skehel J. (2020a). Structural transitions in influenza haemagglutinin at membrane fusion pH. *Nature* 583 150–153. 10.1038/s41586-020-2333-6 32461688 PMC7116728

[B4] Benton D. J. Wrobel A. Xu P. Roustan C. Martin S. Rosenthal P. (2020b). Receptor binding and priming of the spike protein of SARS-CoV-2 for membrane fusion. *Nature* 588 327–330. 10.1038/s41586-020-2772-0 32942285 PMC7116727

[B5] Berg T. Gjöen T. Bakke O. (1995). Physiological functions of endosomal proteolysis. *Biochem. J.* 307:326. 10.1042/bj3070313 7733863 PMC1136650

[B6] Biasin M. Bianco A. Pareschi G. Cavalleri A. Cavatorta C. Fenizia C. (2021). UV-C irradiation is highly effective in inactivating SARS-CoV-2 replication. *Sci. Rep.* 11:6260. 10.1038/s41598-021-85425-w 33737536 PMC7973506

[B7] Biryukov J. Boydston J. Dunning R. Yeager J. Wood S. Ferris A. (2021). SARS-CoV-2 is rapidly inactivated at high temperature. *Environ. Chem. Lett.* 19 1773–1777. 10.1007/s10311-021-01187-x 33551702 PMC7856623

[B8] Böttcher C. Ludwig K. Herrmann A. Van Heel M. Stark H. (1999). Structure of influenza haemagglutinin at neutral and at fusogenic pH by electron cryo-microscopy. *FEBS Lett.* 463 255–259. 10.1016/S0014-5793(99)01475-1 10606732

[B9] Bouvier N. M. Palese P. (2008). The biology of influenza viruses. *Vaccine* 26(Suppl. 4), D49–D53. 10.1016/j.vaccine.2008.07.039 19230160 PMC3074182

[B10] Brandenburg B. Zhuang X. (2007). Virus trafficking – learning from single-virus tracking. *Nat. Rev. Microbiol.* 5 197–208. 10.1038/nrmicro1615 17304249 PMC2740720

[B11] Cabrera-Garcia D. Bekdash R. Abbott G. Yazawa M. Harrison N. (2021). The envelope protein of SARS-CoV-2 increases intra-Golgi pH and forms a cation channel that is regulated by pH. *J. Physiol.* 599 2851–2868. 10.1113/JP281037 33709461 PMC8251088

[B12] Cady S. Luo W. Hu F. Hong M. (2009). Structure and function of the influenza A M2 proton channel. *Biochemistry* 48 7356–7364. 10.1021/bi9008837 19601584 PMC2879269

[B13] Caffrey M. Lavie A. (2021). pH-Dependent mechanisms of influenza infection mediated by hemagglutinin. *Front. Mol. Biosci.* 8:777095. 10.3389/fmolb.2021.777095 34977156 PMC8718792

[B14] Calder L. Wasilewski S. Berriman J. Rosenthal P. (2010). Structural organization of a filamentous influenza A virus. *Proc. Natl. Acad. Sci. U S A.* 107 10685–10690. 10.1073/pnas.1002123107 20498070 PMC2890793

[B15] Carr C. Chaudhry C. Kim P. (1997). Influenza hemagglutinin is spring-loaded by a metastable native conformation. *Proc. Natl. Acad. Sci. U S A.* 94 14306–14313. 10.1073/pnas.94.26.14306 9405608 PMC24954

[B16] Chang P. Sealy J. Sadeyen J. Bhat S. Lukosaityte D. Sun Y. (2020). Immune escape adaptive mutations in the H7N9 avian influenza hemagglutinin protein increase virus replication fitness and decrease pandemic potential. *J. Virol.* 94 e216–e220. 10.1128/JVI.00216-20 32699084 PMC7495387

[B17] Chen H. Wu Y. Voth G. (2007). Proton transport behavior through the influenza A M2 channel: insights from molecular simulation. *Biophys. J.* 93 3470–3479. 10.1529/biophysj.107.105742 17693473 PMC2072055

[B18] Chen J. Lee K. Steinhauer D. Stevens D. Skehel J. Wiley D. (1998). Structure of the hemagglutinin precursor cleavage site, a determinant of influenza pathogenicity and the origin of the labile conformation. *Cell* 95 409–417. 10.1016/S0092-8674(00)81771-7 9814710

[B19] Chen Y. Li M. X. Lu G. D. Shen H. M. Zhou J. (2021). Hydroxychloroquine/Chloroquine as therapeutics for COVID-19: truth under the Mystery. *Int. J. Biol. Sci.* 17 1538–1546. 10.7150/ijbs.59547 33907517 PMC8071775

[B20] Chizhmakov I. Geraghty F. Ogden D. Hayhurst A. AntoniouM Hay A. (1996). Selective proton permeability and pH regulation of the influenza virus M2 channel expressed in mouse erythroleukaemiacells. *J. Physiol.* 494 329–336. 10.1113/jphysiol.1996.sp021495 8841994 PMC1160637

[B21] Chizhmakov I. Ogden D. Geraghty F. Hayhurst A. Skinner A. Betakova T. (2003). Differences in conductance of M2 protonchannels of two influenza viruses at low and high pH. *J. Physiol.* 546 427–438. 10.1113/jphysiol.2002.028910 12527729 PMC2342522

[B22] Cross K. Langley W. Russell R. Skehel J. Steinhauer D. (2009). Composition and functions of the influenza fusion peptide. *Protein Pept. Lett.* 16 766–778. 10.2174/092986609788681715 19601906

[B23] Cutler D. Summers L. (2020). The COVID-19 pandemic and the $16 trillion virus. *JAMA* 324 1495–1496. 10.1001/jama.2020.19759 33044484 PMC7604733

[B24] Dabisch P. Schuit M. Herzog A. Beck K. Wood S. Krause M. (2020). The influence of temperature, humidity, and simulated sunlight on the infectivity of SARS-CoV-2 in aerosols. *Aerosol. Sci. Technol.* 55 142–153. 10.1080/02786826.2020.1829536 PMC10698725 38077296

[B25] de Vries E. Tscherne D. Wienholts M. Cobos-Jiménez V. Scholte F. García-Sastre A. (2011). Dissection of the influenza A virus endocytic routes reveals macropinocytosis as an alternative entry pathway. *PLoS Pathogens* 7:e1001329. 10.1371/journal.ppat.1001329 21483486 PMC3068995

[B26] Dehrmann F. Coetzer T. Pike R. Dennison C. (1995). Mature cathepsin L is substantially active in the ionic milieu of the extracellular medium. *Arch. Biochem. Biophys.* 324 93–98. 10.1006/abbi.1995.9924 7503566

[B27] Dehrmann F. M. Elliott E. Dennison C. (1996). Reductive activation markedly increases the stability of cathepsins B and L to extracellular ionic conditions. *Biol. Chem. Hoppe-Seyler.* 377 391–394. 10.1515/bchm3.1996.377.6.391 8839985

[B28] Dennison C. (2003). *A Guide to Protein Isolation*, 2nd Edn. Netherlands: Kluwer Academic Publishers. 10.1007/978-94-017-0269-0

[B29] Di Trani L. Savarino A. Campitelli L. Norelli S. Puzelli S. D’Ostilio D. (2007). Different pH requirements are associated with divergent inhibitory effects of chloroquine on human and avian influenza A viruses. *Virol. J.* 4:39. 10.1186/1743-422X-4-39 17477867 PMC1878474

[B30] Dolan K. Dutta M. Kern D. Kotecha A. Voth G. Brohawn S. (2022). Structure of SARS-CoV-2 M protein in lipid nanodiscs. *eLife* 11:e81702. 10.7554/eLife.81702 36264056 PMC9642992

[B31] Dou D. Revol R. Östbye H. Wang H. Daniels R. (2018). Influenza A virus cell entry, replication, virion assembly and movement. *Front. Immunol.* 9:1581. 10.3389/fimmu.2018.01581 30079062 PMC6062596

[B32] Fendrick A. M. Monto A. S. Nightengale B. Sarnes M. (2003). The economic burden of non-influenzarelated viral respiratory tract infection in the United States. *Arch. Intern. Med.* 163 487–494. 10.1001/archinte.163.4.487 12588210

[B33] Fontana J. Cardone G. Heymann J. Winkler D. Steven A. (2012). Structural changes in influenza virus at low pH characterized by cryo-electron tomography. *J. Virol.* 86 2919–2929. 10.1128/JVI.06698-11 22258245 PMC3302323

[B34] Galloway S. Reed M. Russell C. Steinhauer D. (2013). Influenza HA subtypes demonstrate divergent phenotypes for cleavage activation and pH of fusion: implications for host range and adaptation. *PLoS Pathog.* 9:e1003151. 10.1371/journal.ppat.1003151 23459660 PMC3573126

[B35] Guaita M. Watters S. Loerch S. (2022). Recent advances and current trends in cryo-electron microscopy. *Curr. Opin. Struct. Biol.* 77:102484. 10.1016/j.sbi.2022.102484 36323134 PMC10266358

[B36] Hansen C. Chaves S. Demont C. Viboud C. (2022). Mortality associated with influenza and respiratory syncytial virus in the US, 1999-2018. *JAMA Network Open* 5:e220527. 10.1001/jamanetworkopen.2022.0527 35226079 PMC8886548

[B37] Höfer C. Di Lella S. Dahmani I. Jungnick N. Bordag N. Bobone S. (2019). Structural determinants of the interaction between influenza A virus matrix protein M1 and lipid membranes. *Biochim Biophys. Acta Biomembr.* 1861 1123–1134. 10.1016/j.bbamem.2019.03.013 30902626

[B38] Hoffmann M. Kleine-Weber H. Schroeder S. Krüger N. Herrler T. Erichsen S. (2020). SARS-CoV-2 cell entry depends on ACE2 and TMPRSS2 and is blocked by a clinically proven protease inhibitor. *Cell* 181 271–280.e8. 10.1016/j.cell.2020.02.052 32142651 PMC7102627

[B39] Hu J. Fu R. Nishimura K. Zhang L. Zhou H. Busath D. (2006). Histidines, heart of the hydrogen ion channel from influenza A virus: toward an understanding of conductance and proton selectivity. *Proc. Natl. Acad. Sci. U S A.* 103 6865–6870. 10.1073/pnas.0601944103 16632600 PMC1458985

[B40] Hu Y. Wen J. Tang L. Zhang H. Zhang X. Li Y. (2003). The M protein of SARS-CoV: basic structural and immunological properties. *Genom. Proteom. Bioinform.* 1 118–130. 10.1016/S1672-0229(03)01016-7 15626342 PMC5172243

[B41] Huang Q. Opitz R. Knapp E. W. Herrmann A. (2002). Protonation and stability of the globular domain of influenzavirus hemagglutinin. *Biophys. J.* 82 1050–1058. 10.1016/S0006-3495(02)75464-7 11806944 PMC1301911

[B42] Huang Y. Yang C. Xu X. Xu W. Liu S. (2020). Structural and functional properties of SARS-CoV-2 spike protein: potential antivirus drug development for COVID-19. *Acta Pharmacol. Sin.* 41 1141–1149. 10.1038/s41401-020-0485-4 32747721 PMC7396720

[B43] Intharathep P. Laohpongspaisan C. Rungrotmongkol T. Loisruangsin A. Malaisree M. Decha P. (2008). How amantadine and rimantadine inhibit proton transport in the M2 protein channel. *J. Mol. Graph Model.* 27 342–348. 10.1016/j.jmgm.2008.06.002 18620883

[B44] Iuliano A. D. Roguski K. Chang H. Muscatello D. Palekar R. Tempia S. (2018). Estimates of global seasonal influenza-associated respiratory mortality: a modelling study. *Lancet* 391 1285–1300. 10.1016/S0140-6736(17)33293-2 29248255 PMC5935243

[B45] Ivanovic T. Rozendaal R. Floyd D. Popovic M. van Oijen A. Harrison S. (2012). Kinetics of proton transport into influenza virions by the viral M2 channel. *PLoS One* 7:e31566. 10.1371/journal.pone.0031566 22412838 PMC3295812

[B46] Jackson C. Farzan M. Chen B. Choe H. (2022). Mechanisms of SARS-CoV-2 entry into cells. *Nat. Rev. Mol. Cell Biol.* 23 3–20. 10.1038/s41580-021-00418-x 34611326 PMC8491763

[B47] James S. L. Abate D. Abate K. Abay S. Abbafati C. Abbasi N. (2018). Global, regional, and national incidence, prevalence, and years lived with disability for 354 diseases and injuries for 195 countries and territories, 1990–2017: a systematic analysis for the Global Burden of Disease Study 2017. *Lancet* 392 1789–1858. 10.1016/S0140-6736(18)32279-7 30496104 PMC6227754

[B48] Kashour Z. Riaz M. Garbati M. AlDosary O. Tlayjeh H. Gerberi D. (2021). Efficacy of chloroquine or hydroxychloroquine in COVID-19 patients: a systematic review and meta-analysis. *J. Antimicrob Chemother.* 76 30–42. 10.1093/jac/dkaa403 33031488 PMC7665543

[B49] Kelly M. Cook J. Brown-Augsburger P. Heinz B. Smith M. Pinto L. (2003). Demonstrating the intrinsic ion channel activity of virally encoded proteins. *FEBS Lett.* 552 61–67. 10.1016/S0014-5793(03)00851-2 12972153

[B50] Kemble G. Bodian D. Rosé J. Wilson I. White J. (1992). Intermonomer disulfide bonds impair the fusion activity of influenza virus hemagglutinin. *J. Virol.* 66 4940–4950. 10.1128/jvi.66.8.4940-4950.1992 1629960 PMC241339

[B51] Kennedy C. D. (1990). Ionic strength and the dissociation of acids. *Biochem. Educ.* 18 35–40. 10.1016/0307-4412(90)90017-I

[B52] Kiss A. L. Botos E. (2009). Endocytosis via caveolae: alternative pathway with distinct cellular compartments to avoid lysosomal degradation? *J. Cell. Mol. Med.* 13 1228–1237. 10.1111/j.1582-4934.2009.00754.x 19382909 PMC4496137

[B53] Koch J. Uckeley Z. Doldan P. Stanifer M. Boulant S. Lozach P. (2021). TMPRSS2 expression dictates the entry route used by SARS-CoV-2 to infect host cells. *EMBO J.* 40:e107821. 10.15252/embj.2021107821 34159616 PMC8365257

[B54] Kreutzberger A. Sanyal A. Saminathan A. Bloyet L. Stumpf S. Liu Z. (2022). SARS-CoV-2 requires acidic pH to infect cells. *Proc. Natl. Acad. Sci. U S A.* 119:e2209514119. 10.1073/pnas.2209514119 36048924 PMC9499588

[B55] Kuo L. Hurst K. Masters P. (2007). Exceptional flexibility in the sequence requirements for coronavirus small envelope protein function. *J. Virol.* 81 2249–2262. 10.1128/JVI.01577-06 17182690 PMC1865940

[B56] Lakadamyali M. Rust M. Zhuang X. (2004). Endocytosis of influenza viruses. *Microbes Infect.* 6 929–936. 10.1016/j.micinf.2004.05.002 15310470 PMC2715838

[B57] Lamb R. A. Choppin P. W. (1983). The structure and replication of influenza virus. *Annu. Rev. Biochem.* 52 467–506. 10.1146/annurev.bi.52.070183.002343 6351727

[B58] Lan Y. He W. Wang G. Wang Z. Chen Y. Gao F. (2022). Potential antiviral strategy exploiting dependence of SARS-CoV-2 replication on lysosome-based pathway. *Int. J. Mol. Sci.* 23:6188. 10.3390/ijms23116188 35682877 PMC9181800

[B59] Lee K. (2010). Architecture of a nascent viral fusion pore. *EMBO J.* 29 1299–1311. 10.1038/emboj.2010.13 20168302 PMC2857459

[B60] Lentz M. R. Webster R. G. Air G. M. (1987). Site-directed mutation of the active site of influenza neuraminidase and implications for the catalytic mechanism. *Biochemistry* 26 5351–5358. 10.1021/bi00391a020 3314986

[B61] Li W. Moore M. Vasilieva N. Sui J. Wong S. Berne M. (2003). Angiotensin-converting enzyme 2 is a functional receptor for the SARS coronavirus. *Nature* 426 450–454. 10.1038/nature02145 14647384 PMC7095016

[B62] Lin T. I. Schroeder C. (2001). Definitive assignment of proton selectivity and attoampere unitary current to the M2 ion channel protein of influenza A virus. *J. Virol.* 75:3647. 10.1128/JVI.75.8.3647-3656.2001 11264354 PMC114856

[B63] Lopez-Leon S. Wegman-Ostrosky T. Perelman C. Sepulveda R. Rebolledo P. Cuapio A. (2021). More than 50 long-term effects of COVID-19: a systematic review and meta-analysis. *Sci. Rep.* 11:16144. 10.1038/s41598-021-95565-8 34373540 PMC8352980

[B64] Luo B. Schaub A. Glas I. Klein L. David S. Bluvshtein N. (2023). Expiratory aerosol pH: the overlooked driver of airborne virus inactivation. *Environ. Sci. Technol.* 57 486–497. 10.1021/acs.est.2c05777 36537693 PMC9835828

[B65] Luzio J. Pryor P. Bright N. (2007). Lysosomes: fusion and function. *Nat. Rev. Mol. Cell Biol.* 8 622–632. 10.1038/nrm2217 17637737

[B66] Maeda T. Kawasaki K. Ohnishi S. (1981). Interaction of influenza virus hemagglutinin with target membrane lipids is a key step in virus-induced hemolysis and fusion at pH 5.2. *Proc. Natl. Acad. Sci. U S A.* 78 4133–4137. 10.1073/pnas.78.7.4133 6945575 PMC319742

[B67] Mair C. Ludwig K. Herrmann A. Sieben C. (2014a). Receptor binding and pH stability - how influenza A virus hemagglutinin affects host-specific virus infection. *Biochim Biophys. Acta* 1838 1153–1168. 10.1016/j.bbamem.2013.10.004 24161712

[B68] Mair C. Meyer T. Schneider K. Huang Q. Veit M. Herrmann A. (2014b). A histidine residue of the influenza virus hemagglutinin controls the pH dependence of the conformational change mediating membrane fusion. *J. Virol.* 88 13189–13200. 10.1128/JVI.01704-14 25187542 PMC4249083

[B69] Mason R. W. Massey S. D. (1992). Surface activation of pro-cathepsin L. *Biochem. Biophys. Res. Commun.* 189 1659–1666. 10.1016/0006-291X(92)90268-P 1482371

[B70] Matlin K. Reggio H. Helenius A. Simons K. (1981). Infectious entry pathway of influenza virus in a canine kidney cell line. *J. Cell Biol.* 91(3 Pt 1), 601–613. 10.1083/jcb.91.3.601 7328111 PMC2112819

[B71] McKimm-Breschkin J. L. (2000). Resistance of influenza viruses to neuraminidase inhibitors – a review. *Antivir. Res.* 47 1–17. 10.1016/S0166-3542(00)00103-0 10930642

[B72] Meng B. Abdullahi A. Ferreira I. Goonawardane N. Saito A. Kimura I. (2022). Altered TMPRSS2 usage by SARS-CoV-2 Omicron impacts infectivity and fusogenicity. *Nature* 603 706–714. 10.1038/s41586-022-04474-x 35104837 PMC8942856

[B73] Mohamed M. Sloane B. (2006). Cysteine cathepsins: multifunctional enzymes in cancer. *Nat. Rev. Cancer* 6 764–775. 10.1038/nrc1949 16990854

[B74] Mould J. Drury J. Frings S. Kaupp U. Pekosz A. Pinto L. (2000a). Permeation and activation of the M2ion channel ofinfluenza A virus. *J. Biol. Chem.* 275 31038–31050. 10.1074/jbc.M003663200 10913133

[B75] Mould J. A. Li H. Dudlak C. Lear J. Pekosz A. Lamb R. (2000b). Mechanism for proton conduction of the M(2) ion channel of influenza A virus. *J. Biol. Chem.* 275 8592–8599. 10.1074/jbc.275.12.8592 10722698

[B76] Mountford C. E. Grossman G. Holmes K. T. O’Sullivan W. J. Hampson A. W. Raison R. L. (1982). Effect of monoclonal anti-neuraminidase antibodies on the kinetic behavior of influenza virus neuraminidase. *Mol. Immunol.* 19 811–816. 10.1016/0161-5890(82)90007-4 7110144

[B77] Msemburi W. Karlinsky A. Knutson V. Aleshin-Guendel S. Chatterji S. Wakefield J. (2023). The WHO estimates of excess mortality associated with the COVID-19 pandemic. *Nature* 613 130–137. 10.1038/s41586-022-05522-2 36517599 PMC9812776

[B78] Neuman B. Kiss G. Kunding A. Bhella D. Baksh M. Connelly S. (2011). A structural analysis of M protein in coronavirus assembly and morphology. *J. Struct. Biol.* 174 11–22. 10.1016/j.jsb.2010.11.021 21130884 PMC4486061

[B79] Nieto-Torres J. L. Verdiá-Báguena C. Jimenez-Guardeño J. M. Regla-Nava J. A. Castaño-Rodriguez C. Fernandez-Delgado R. (2015). Severe acute respiratory syndrome coronavirus E protein transports calcium ions and activates the NLRP3 inflammasome. *Virology* 485 330–339. 10.1016/j.virol.2015.08.010 26331680 PMC4619128

[B80] Noton S. Medcalf E. Fisher D. Mullin A. Elton D. Digard P. (2007). Identification of the domains of the influenza A virus M1 matrix protein required for NP binding, oligomerization and incorporation into virions. *J. General Virol.* 88(Pt 8), 2280–2290. 10.1099/vir.0.82809-0 17622633 PMC2884976

[B81] Örd M. Faustova I. Loog M. (2020). The sequence at Spike S1/S2 site enables cleavage by furin and phospho-regulation in SARS-CoV2 but not in SARS-CoV1 or MERS-CoV. *Sci. Rep.* 10:16944. 10.1038/s41598-020-74101-0 33037310 PMC7547067

[B82] Ouzounis C. A. (2020). A recent origin of Orf3a from M protein across the coronavirus lineage arising by sharp divergence. *Comput. Struct. Biotechnol. J.* 18 4093–4102. 10.1016/j.csbj.2020.11.047 33363705 PMC7749296

[B83] Padilla-Parra S. Matos P. M. Kondo N. Marin M. Santos N. Melikyan G. B. (2012). Quantitative imaging of endosome acidification and single retrovirus fusion with distinct pool of early endosomes. *Proc. Natl. Acad. Sci. U S A.* 109 17627–17632. 10.1073/pnas.1211714109 23047692 PMC3491504

[B84] Palese P. Tobita K. Ueda M. Compans R. W. (1974). Characterization of temperature sensitive influenza virus mutants defective in neuraminidase. *Virology* 61 397–410. 10.1016/0042-6822(74)90276-1 4472498

[B85] Peacock T. P. Goldhill D. H. Zhou J. Baillon L. Frise R. Swann O. (2021). The furin cleavage site in the SARS-CoV-2 spike protein is required for transmission in ferrets. *Nat. Microbiol.* 6 899–909. 10.1038/s41564-021-00908-w 33907312 PMC7619196

[B86] Peukes J. Xiong X. Erlendsson S. Qu K. Wan W. Calder L. (2020). The native structure of the assembled matrix protein 1 of influenza A virus. *Nature* 587 495–498. 10.1038/s41586-020-2696-8 32908308 PMC7116405

[B87] Phongphanphanee S. Rungrotmongkol T. Yoshida N. Hannongbua S. Hirata F. (2010). Proton transport through the influenza A M2 channel: three-dimensional reference interaction site model study. *J. Am. Chem. Soc.* 132 9782–9788. 10.1021/ja1027293 20578761

[B88] Pielak R. M. Chou J. J. (2011). Influenza M2 proton channels. *Biochimica Biophys. Acta* 1808 522–529. 10.1016/j.bbamem.2010.04.015 20451491 PMC3108042

[B89] Pinto L. Dieckmann G. Gandhi C. Papworth C. Braman J. Shaughnessy M. (1997). A functionally defined model for the M2 proton channel of influenza A virus suggests a mechanism for its ion selectivity. *Proc. Natl. Acad. Sci. U S A.* 94 11301–11306. 10.1073/pnas.94.21.11301 9326604 PMC23448

[B90] Pinto L. Holsinger L. Lamb R. (1992). Influenza virus M2 protein has ion channel activity. *Cell* 69 517–528. 10.1016/0092-8674(92)90452-I 1374685

[B91] Rice A. Haldar S. Wang E. Blank P. Akimov S. Galimzyanov T. (2022). Planar aggregation of the influenza viral fusion peptide alters membrane structure and hydration, promoting poration. *Nat. Commun.* 13:7336. 10.1038/s41467-022-34576-z 36470871 PMC9722698

[B92] Ruigrok R. W. Calder L. J. Wharton S. A. (1989). Electron microscopy of the influenza virus submembranal structure. *Virology* 173 311–316. 10.1016/0042-6822(89)90248-1 2815585

[B93] Rust M. J. Lakadamyali M. Zhang F. Zhuang X. (2004). Assembly of endocytic machinery around individual influenza viruses during viral entry. *Nat. Struct. Mol. Biol.* 11 567–573. 10.1038/nsmb769 15122347 PMC2748740

[B94] Sachs J. Karim S. Aknin L. Allen J. Brosbøl K. Colombo F. (2022). The Lancet Commission on lessons for the future from the COVID-19 pandemic. *Lancet* 400 1224–1280. 10.1016/S0140-6736(22)01585-9 36115368 PMC9539542

[B95] Sakaguchi T. Leser G. Lamb R. (1996). The ion channel activity of the influenza virus M2 protein affects transport through the Golgi apparatus. *J. Cell Biol.* 133 733–747. 10.1083/jcb.133.4.733 8666660 PMC2120830

[B96] Schnell J. Chou J. (2008). Structure and mechanism of the M2 proton channel of influenza A virus. *Nature* 451 591–595. 10.1038/nature06531 18235503 PMC3108054

[B97] Schoeman D. Fielding B. (2019). Coronavirus envelope protein: current knowledge. *Virol. J.* 16:69. 10.1186/s12985-019-1182-0 31133031 PMC6537279

[B98] Selzer L. Su Z. Pintilie G. Chiu W. Kirkegaard K. (2020). Full-length three-dimensional structure of the influenza A virus M1 protein and its organization into a matrix layer. *PLoS Biol.* 18:e3000827. 10.1371/journal.pbio.3000827 32997652 PMC7549809

[B99] Shang C. Zhuang X. Zhang H. Li Y. Zhu Y. Lu J. (2021). Inhibitors of endosomal acidification suppress SARS-CoV-2 replication and relieve viral pneumonia in hACE2 transgenic mice. *Virol. J.* 18:46. 10.1186/s12985-021-01515-1 33639976 PMC7914043

[B100] Shi T. McAllister D. O’Brien K. Simoes E. Madhi S. Gessner B. (2017). Global, regional, and national disease burden estimates of acute lower respiratory infections due to respiratory syncytial virus in young children in 2015: a systematic review and modelling study. *Lancet* 390 946–958. 10.1016/S0140-6736(17)30938-8 28689664 PMC5592248

[B101] Shirato K. Kanou K. Kawase M. Matsuyama S. (2017). Clinical isolates of human coronavirus 229E bypass the endosome for cell entry. *J. Virol.* 91:JVI.01387-16. 10.1128/JVI.01387-16 27733646 PMC5165181

[B102] Shirato K. Kawase M. Matsuyama S. (2018). Wild-type human coronaviruses prefer cell-surface TMPRSS2 to endosomal cathepsins for cell entry. *Virology* 517 9–15. 10.1016/j.virol.2017.11.012 29217279 PMC7112029

[B103] Shtykova E. V. Dadinova L. A. Fedorova N. V. Golanikov A. Bogacheva E. Ksenofontov A. (2017). Influenza virus Matrix Protein M1 preserves its conformation with pH, changing multimerization state at the priming stage due to electrostatics. *Sci. Rep.* 7:16793. 10.1038/s41598-017-16986-y 29196731 PMC5711849

[B104] Sriwilaijaroen N. Suzuki Y. (2012). Molecular basis of the structure and function of H1 hemagglutinin of influenza virus. *Proc. Japan Acad. Ser B Phys. Biol. Sci.* 88 226–249. 10.2183/pjab.88.226 22728439 PMC3410141

[B105] Staschke K. Hatch S. Tang J. Hornback W. Munroe J. Colacino J. (1998). Inhibition of influenza virus hemagglutinin-mediated membrane fusion by a compound related to podocarpic acid. *Virology* 248 264–274. 10.1006/viro.1998.9273 9721235

[B106] Stegmann T. Booy F. Wilschut J. (1987). Effects of low pH on influenza virus. Activation and inactivation of the membrane fusion capacity of the hemagglutinin. *J. Biol. Chem.* 262 17744–17749. 10.1016/S0021-9258(18)45442-7 3693369

[B107] Takahashi T. Suzuki T. (2015). Low-pH stability of influenza A virus sialidase contributing to virus replication and pandemic. *Biol. Pharm. Bull.* 38 817–826. 10.1248/bpb.b15-00120 26027822

[B108] Tortorici M. Walls A. Lang Y. Wang C. Li Z. Koerhuis D. (2019). Structural basis for human coronavirus attachment to sialic acid receptors. *Nat. Struct. Mol. Biol.* 26 481–489. 10.1038/s41594-019-0233-y 31160783 PMC6554059

[B109] Trost J. Wang W. Liang B. Galloway S. Agbogu E. Byrd-Leotis L. (2019). A conserved histidine in Group-1 influenza subtype hemagglutinin proteins is essential for membrane fusion activity. *Virology* 536 78–90. 10.1016/j.virol.2019.08.005 31401467 PMC6733629

[B110] Turk B. Dolenc I. Turk V. Bieth J. (1993). Kinetics of the pH-induced inactivation of human cathepsin L. *Biochemistry* 32 375–380. 10.1021/bi00052a046 7678196

[B111] Van Dyke R. W. (1995). “Acidification of lysosomes and endosomes,” in *Biology of the Lysosome, Subcellular Biochemistry*, eds Lloyd J. B. Mason R. W. (New York, NY: Plenum Press), 331360. 10.1007/978-1-4615-5833-0_10

[B112] Venkataraman P. Lamb R. A. Pinto L. H. (2005). Chemical rescue of histidine selectivity filter mutants of the M2 ion channel of influenza A virus. *J. Biol. Chem.* 280 21463–21472. 10.1074/jbc.M412406200 15784624

[B113] Verdiá-Báguena C. Nieto-Torres J. L. Alcaraz A. DeDiego M. L. Torres J. Aguilella V. M. (2012). Coronavirus E protein forms ion channels with functionally and structurally-involved membrane lipids. *Virology* 432 485–494. 10.1016/j.virol.2012.07.005 22832120 PMC3438407

[B114] Wallabregue A. Moreau D. Sherin P. Moneva Lorente P. Jarolímová Z. Bakker E. (2016). Selective imaging of late endosomes with a pH-Sensitive diazaoxatriangulene fluorescent probe. *J. Am. Chem. Soc.* 138 1752–1755. 10.1021/jacs.5b09972 26799309

[B115] Walls A. C. Tortorici M. Snijder J. Xiong X. Bosch B. Rey F. (2017). Tectonic conformational changes of a coronavirus spike glycoprotein promote membrane fusion. *Proc. Natl Acad. Sci. U S A.* 114 11157–11162. 10.1073/pnas.1708727114 29073020 PMC5651768

[B116] Wang C. Lamb R. Pinto L. (1995). Activation of the M2 ion channel of influenza virus: a role for the transmembrane domain histidine residue. *Biophys. J.* 69 1363–1371. 10.1016/S0006-3495(95)80003-2 8534806 PMC1236366

[B117] Xia B. Shen X. He Y. Pan X. Liu F. Wang Y. (2021). SARS-CoV-2 envelope protein causes acute respiratory distress syndrome (ARDS)-like pathological damages and constitutes an antiviral target. *Cell Res.* 31 847–860. 10.1038/s41422-021-00519-4 34112954 PMC8190750

[B118] Zaraket H. Bridges O. Russell C. (2013). The pH of activation of the hemagglutinin protein regulates H5N1 influenza virus replication and pathogenesis in mice. *J. Virol.* 87 4826–4834. 10.1128/JVI.03110-12 23449784 PMC3624295

[B119] Zebedee S. Lamb R. (1988). Influenza A virus M2 protein: monoclonal antibody restriction of virus growth and detection of M2 in virions. *J. Virol.* 62 2762–2772. 10.1128/jvi.62.8.2762-2772.1988 2455818 PMC253710

[B120] Zhang K. Wang Z. Liu X. Yin C. Basit Z. Xia B. (2012). Dissection of influenza A virus M1 protein: pH-dependent oligomerization of N-terminal domain and dimerization of C-terminal domain. *PLoS One* 7:e37786. 10.1371/journal.pone.0037786 22655068 PMC3360003

[B121] Zhang Z. Nomura N. Muramoto Y. Ekimoto T. Uemura T. Liu K. (2022). Structure of SARS-CoV-2 membrane protein essential for virus assembly. *Nat. Commun.* 13:4399. 10.1038/s41467-022-32019-3 35931673 PMC9355944

[B122] Zhao M. Yang W. Yang F. Zhang L. Huang W. Hou W. (2021). Cathepsin L plays a key role in SARS-CoV-2 infection in humans and humanized mice and is a promising target for new drug development. *Sig. Transduct. Target Ther.* 6:134. 10.1038/s41392-021-00558-8 33774649 PMC7997800

[B123] Zhou Y. Wu C. Huang N. (2014). Exploring the early stages of the pH-induced conformational change of influenza hemagglutinin. *Proteins* 82 2412–2428. 10.1002/prot.24606 24854389

[B124] Zucs P. Buchholz U. Haas W. Uphoff H. (2005). Influenza associated excess mortality in Germany, 1985-2001. *Emerg. Themes Epidemiol.* 2:6. 10.1186/1742-7622-2-6 PMC1188065 15969758

